# Neuropathological findings in COVID-19 vs. non-COVID-19 acute respiratory distress syndrome—A case-control study

**DOI:** 10.3389/fneur.2023.1283698

**Published:** 2023-12-22

**Authors:** Mariyam Humayun, Lucy Zhang, Thomas D. Zaikos, Nivedha Kannapadi, Jose I. Suarez, David N. Hager, Juan C. Troncoso, Sung-Min Cho

**Affiliations:** ^1^Division of Neuroscience Critical Care, Departments of Neurology, Neurosurgery, Anesthesiology and Critical Care Medicine, Johns Hopkins University School of Medicine, Baltimore, MD, United States; ^2^Inova Health System, Falls Church, VA, United States; ^3^Neuropathology Division, Department of Pathology, Johns Hopkins University School of Medicine, Baltimore, MD, United States; ^4^Division of Pulmonary and Critical Care Medicine, Department of Medicine, Johns Hopkins University School of Medicine, Baltimore, MD, United States

**Keywords:** acute respiratory distress syndrome, COVID-19, acute brain injury, neuropathology, brain autopsy

## Abstract

Acute brain injury (ABI) and neuroinflammation is reported in COVID-19 and acute respiratory distress syndrome (ARDS). It remains unclear if COVID-19 plays an independent role in development of ABI compared to those with non-COVID-19 ARDS. We aimed to evaluate if COVID-19 ARDS is associated with higher risk and specific patterns of ABI compared to non-COVID-19 ARDS. We conducted an age and sex matched case-control autopsy study at a tertiary academic center. Ten patients with COVID-19 ARDS were matched to 20 non-COVID-19 ARDS patients. Baseline demographics were comparable between the two groups including severity of ARDS (*p* = 0.3). The frequency of overall ABI (70 vs. 60%), infratentorial ABI (40 vs. 25%), ischemic infarct (40 vs. 25%), intracranial hemorrhage (30 vs. 35%), and hypoxic-ischemic brain injury (30 vs. 35%) was similar between COVID-19 and non-COVID-19 ARDS patients, respectively (*p* > 0.05). Intracapillary megakaryocytes were exclusively seen in 30% of COVID-19 patients. Overall, frequency and pattern of ABI in COVID-19 ARDS was comparable to non-COVID-19.

## 1 Introduction

SARS-CoV-2 is a primary respiratory pathogen that has been linked to a range of cerebrovascular, cognitive, and neuroimmunological conditions (1). A spectrum of neuropathological features have been described in autopsy studies of COVID-19 patients (2). Hypoxic ischemic injury, microglial activation, lymphoid inflammation, and ischemic infarcts are some of the neuropathological features described in this population (3, 4). A study evaluating hippocampal changes in post COVID syndrome showed alterations in microstructural integrity, functional connectivity and concentration of certain histological biomarkers including GFAP and MOG (5). Overall, neuropathological findings in COVID-19 can be described as varied and heterogenous, with unclear causation to acute brain injury.

Acute respiratory distress syndrome (ARDS), a known complication of COVID-19, is characterized by extensive inflammation, hypoxemia, and use of mechanical ventilation which can lead to secondary brain injury (6). A high frequency of acute brain injury (ABI) has also been described in non-COVID-19 patients with ARDS; the most common types of ABI reported are intracranial hemorrhage and hypoxic-ischemic brain injury (HIBI) (6). Neuroinflammatory changes recorded on brain pathology in COVID-19 are similar to neuropathological changes described in human and animal models of severe influenza (7, 8). These findings suggest that severe respiratory disease may be independently linked with brain injury.

COVID-19 has been associated with neuro-inflammatory changes, and it was postulated that these findings were more pronounced in the brainstem and cerebellum (9). Microglial activation in the infratentorial region is commonly noted in COVID-19 patients (7, 9). Infiltration of cytotoxic T-cells and microglial nodules is also more frequent in the brainstem and cerebellum (3, 7, 9). It remains unclear if these inflammatory changes translate into ABI and if the regional tropism leads to a higher ABI in certain central nervous system locations in COVID-19 patients that is different from non-COVID-19 ARDS.

Our study aimed to compare the frequency and patterns of ABI among patients with COVID-19 ARDS and non-COVID-19 ARDS in a neuropathology study. We hypothesized that COVID-19 ARDS patients are at higher risk of developing ABI with a regional preference for infratentorial locations.

## 2 Materials and methods

### 2.1 Subjects

This is a retrospective case-control study. We identified consecutive COVID-19-associated ARDS patients (*n* = 10) from March 2019 to June 2022 from the autopsy registry at a single institution. Adult patients >18 years of age, who tested positive for SARS-CoV-2 virus using the nucleic acid amplification technique and met the Berlin definition of ARDS were included (10). Non-COVID-19 ARDS controls (*n* = 20) were also identified from the institutional autopsy registry from an era preceding the COVID-19 pandemic (February 2008 to December 2018). Cases and controls were matched based on age and sex in a 1:2 manner. Due to the relatively restrictive inclusion criteria of presence of ARDS, age was not able to be perfectly matched for each case. However, matched control cases between the above period were identified based on the those that met all criteria and had an age closest to their counterpart COVID-19 case. Differences in age between COVID-19 cases and control cases ranged between 0 and 16 years. The mean and median age differences of our study cohorts were 3.9 years and 2 years, respectively (data not shown). Patients were not matched for post-mortem interval (PMI) defined as the duration, in hours, from declaration of death to performance of autopsy. No IRB approval was required since this was an autopsy study and did not involve contact with human subjects.

### 2.2 Clinical data

Clinical data including patient demographics, medical history, and ARDS characteristics were collected from chart review. All patients were admitted to an intensive care unit and required mechanical ventilation. Sequential Organ Failure Assessment (SOFA) scores were calculated for each patient and the highest score within 24 h of intubation or admission to ICU was included for analysis. Similarly, ARDS severity on day of intubation was defined using the PaO_2_/FIO_2_ ratio as per the Berlin criteria: mild (200 mmHg < PaO_2_/FIO_2_ ≤ 300 mmHg), moderate (100 mmHg < PaO_2_/FIO_2_ ≤ 200 mmHg), and severe (PaO_2_/FIO_2_ ≤ 100 mmHg) (10). Use of various ARDS therapeutics/interventions was also assessed.

### 2.3 Neuropathology data

Brain autopsies were performed by board-certified neuropathologists according to standard procedures. Reports of gross and microscopic evaluations were reviewed. Slides of brain tissue were not re-examined for the purpose of this study. ABI was defined as the presence of acute/subacute infarction, intracranial hemorrhage (which includes subarachnoid, intraparenchymal, and/or subdural hemorrhage), brain edema and HIBI on autopsy. ABI was also evaluated at a regional level comparing supratentorial and infratentorial lesions. HIBI was further classified as focal, multifocal, and global depending on the extent of injury. Petechial hemorrhages were defined as those seen on macroscopic examination, while microhemorrhages visualized at a microscopic level.

### 2.4 Statistical analysis

Statistical analysis was conducted using STATA version 17.0. Data are reported as percentages and medians with interquartile ranges. Given the preliminary nature of the study, we did not undertake sample size or power calculation. We used the two sample Wilcoxon rank-sum test to compare continuous variables and Chi-square test for all categorical variables. *P-*value < 0.05 was considered statistically significant.

## 3 Results

We identified 10 COVID-19 associated ARDS patients, which were age- and sex-matched to 20 non-COVID-19 ARDS patients. Baseline demographics and clinical characteristics were similar between the groups (Table 1).

**Table 1:** Demographic and disease characteristics of patients with and without COVID-19.

	**COVID-19 ARDS (*n =* 10)**	**Non-COVID-19 ARDS (*n =* 20)**	***P*-value**
Age, median (IQR)	65.5 (56–80)	64 (56–72)	0.71
Male, *n* (%)	7 (70%)	14 (70%)	1.00
Race, *n* (%)			0.96
White	5 (50%)	11 (55%)	
Black	4 (40%)	7 (35%)	
Hispanic	1 (10%)	2 (10%)	
**Past Medical History**			
HTN, *n* (%)	8 (80%)	13 (65%)	0.40
DM, *n* (%)	7 (70%)	7 (35%)	0.70
Coronary artery disease, *n* (%)	3 (30%)	5 (25%)	0.77
HFrEF, *n* (%)	1 (10%)	1 (5%)	0.60
COPD/Asthma, *n* (%)	0 (0%)	3 (15%)	0.197
CKD *n* (%)	3 (30%)	1 (5%)	0.06
Immunocompromised, *n* (%)	1 (10%)	4 (20%)	0.50
History of stroke, *n* (%)	1 (10%)	4 (20%)	0.50
**ARDS characteristics**			
Severity (PaO2/FiO2 ratio)			0.30
Mild	0	2 (10%)	
Moderate	0	0	
Severe	10 (100%)	18 (90%)	
Duration of mechanical ventilation, median (IQR)–days	8 (2–17)	8 (2–14)	0.71
ICU length of stay, median (IQR)–days	11.5 (7–18)	10.5 (5–19.5)	0.79
SOFA Score, median (IQR)	9.5 (6–13)	11.5 (9.5–14)	0.13
Use of paralytics, *n* (%)	3 (30%)	4 (20%)	0.542
Use of inhaled vasodilators, *n* (%)	3 (30%)	0	
Proning, *n* (%)	4 (40%)	1 (5%)	0.02
ECMO, *n* (%)	0	1 (5%)	

SD, Standard deviation; HTN, Hypertension; DM, Diabetes Mellitus; COPD, Chronic obstructive pulmonary disease; HFrEF, Heart failure with reduced ejection fraction; CKD, Chronic kidney disease; ARDS, Acute respiratory distress Syndrome; SOFA, Sequential Organ Failure assessment; ECMO, extracorporeal membrane oxygenation.

### 3.1 ARDS characteristics

All patients in the COVID-19 group and 90% of the non-COVID-19 group had severe ARDS. Median SOFA score at time of intubation for COVID-19 vs. non-COVID-19 group was 9.5 (6–13) and 11.5 (9.5–14) (*p* = 0.13), respectively. On assessment of ARDS therapeutics, there was no difference in use of paralytics and extracorporeal membrane oxygenation (ECMO) between the two groups, though inhaled vasodilators (nitrous oxide) and prone positioning were utilized more frequently in the COVID-19 ARDS group.

### 3.2 Neuropathology data

PMI for autopsy performance was significantly longer for COVID-19 ARDS patients (33.5 vs. 19 hours, *p* = 0.04). The frequency of ABI in all ARDS patients was 63% (19/30). Seven patients (70%) in the COVID-19 group had ABI, compared to 12 patients (60%) in the non-COVID-19 group (*p* = 0.60).

**Figure 1:**
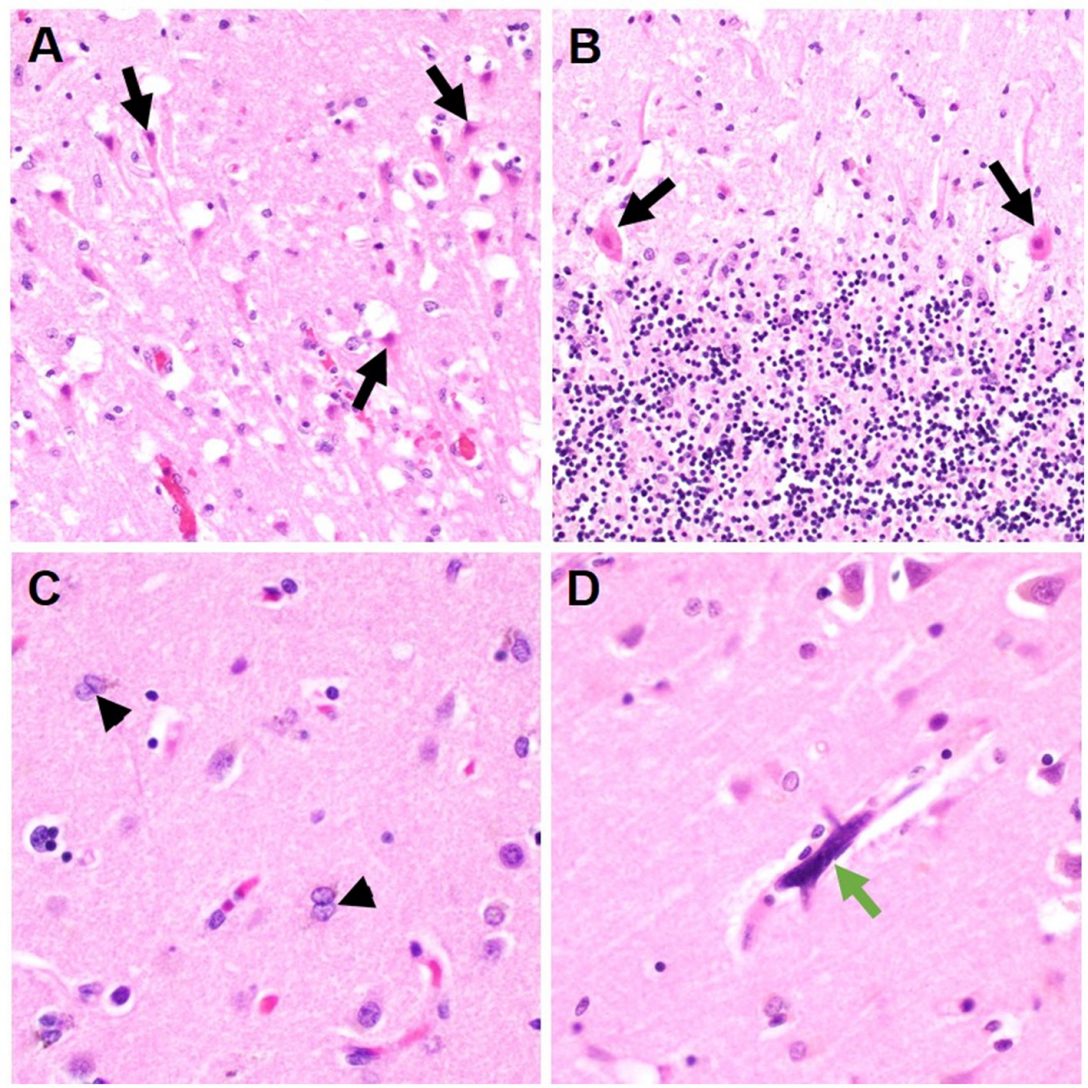
Representative histologic brain autopsy findings in patients with COVID-19 and non-COVID-19 ARDS. Acute hypoxic ichemic brain injury was commonly seen including in susceptible regions such as the hippocampus [**(A)**; black arrows] and Purkinje cell layer of the cerebellar folia [**(B)**; black arrows]. In a subset of cases, Alzheimer's type II astrocytes were identified; a reactive finding associated with systemic metabolic derangements [**(C)**; arrowheads]. Unique to cases of COVID-19 ARDS, scattered intracapilary megakaryocytes were identified in 30% of COVID-19 cases [**(D)**; green arrow].

Location of ABI (infratentorial vs. supratentorial) was not statistically different between the groups. Ischemic infarcts were seen in 40 vs. 25%, hemorrhagic lesions noted in 30 vs. 35%, and HIBI in 30 vs. 35% of COVID-19 and non-COVID ARDS examination, respectively (Table 2). There was no difference in the frequency of cerebral edema, brain herniation, vasculopathy, and gliosis between the COVID-19 and non-COVID-19 ARDS groups. However, neocapillary megakaryotes were exclusively seen in 30% of COVID-19 associated ARDS patients (Figure 1).

**Table 2:** Neuro-pathology findings in patients with and without COVID-19.

	**COVID-19 ARDS (*n =* 10)**	**Non-COVID-19 ARDS (*n =* 20)**	***P*-value**
Brain weight, median (IQR) –grams	1,310 (1,250–1,390)	1,285 (1,225–1,395)	0.77
PMI interval, median (IQR) –hours	33.5 (23–55)	19 (10.5–37.5)	0.04
ABI, *n* (%)	7 (70%)	12 (60%)	0.60
Infratentorial ABI, *n* (%)	4 (40%)	5 (25%)	0.40
HIBI, *n* (%)	3 (30%)	7 (35%)	0.78
Focal	2 (20%)	4 (20%)	
Multifocal	0	1 (5%)	
Diffuse	1 (10%)	2 (10%)	
Ischemic infarct, *n* (%)	4 (40%)	5 (25%)	0.40
Acute	1 (10%)	3 (15%)	0.42
Subacute	1 (10%)	0	
Chronic	2 (20%)	2 (20%)	
Intracranial hemorrhage, *n* (%)^*^	3 (30%)	7 (35%)	0.78
SAH	3 (30%)	1 (5%)	0.42
IPH	1 (10%)	2 (10%)	
Dural	0	1 (5%)	
Petechial	0	2 (10%)	
Microbleed	0	1 (5%)	
Brain Edema, *n* (%)	2 (20%)	2 (10%)	0.45
Intracapillary megakaryocytes, *n* (%)	3 (30%)	0	
Vasculopathy, *n* (%)	6 (60%)	5 (25%)	0.06
Herniation, *n* (%)	2 (20%)	1(5%)	0.20
Gliosis, *n* (%)	2 (20%)	2 (10%)	0.45
Type 2 astrocytes, *n* (%)	1 (10%)	2 (10%)	1.00

ABI, acute brain injury; PMI, post mortem interval; HIBI, Hypoxic ischemic brain injury; SAH, subarachnoid hemorrhage; IPH, intraparenchymal hemorrhage. ^*^One patient in the COVID-19 group had both SAH and IPH present on pathological examination.

## 4 Discussion

A myriad of neurological symptoms has been associated with COVID-19. Thus, we conducted the first age- and sex-matched neuropathology study of COVID-19 ARDS cases to controls to investigate if SARS-CoV-2 confers a higher risk of ABI in COVID-19 vs. non-COVID-19 ARDS. Our study showed no difference in the frequency of ABI on postmortem examination of COVID-19 and non-COVID-19 ARDS patients.

The high frequency of ABI in both groups can potentially be a complication of severe ARDS, and not the specific etiology of lung injury. The brain-lung crosstalk, along with systemic inflammation and hypoxemia, is often offered as an explanation for neurological complications seen in ARDS (6). Prior clinical and autopsy studies have documented neurological complications in non-COVID-19 ARDS (6). In a matched case-control study, Shoskes et al. did not find a difference in ischemic and hemorrhagic ABI on brain MRI between COVID-19 and non-COVID-19 ARDS patients, with the exception of disseminated hemorrhagic leukoencephalopathy being more common in COVID-19 ARDS patients (11). Similarly, our study does not show a difference in ABI between patients that died of COVID-19 and non-COVID-19 ARDS.

Others have suggested that COVID-19 may have a predilection for the brainstem, with autopsy studies documenting pronounced neuro-inflammatory changes in COVID-19 infratentorial specimens (9). Immune dysfunction and vascular activation seen in these critical areas of the central nervous system may be responsible for some of the short- and long-term neurological symptoms (12). Although limited to basic histopathologic examination, our study found no differences between patients with ARDS due to COVID-19 vs. other causes. Potential COVID-19-specific brainstem pathology might not be easily identifiable by standard brain autopsies and could require ancillary testing. Therefore, whether these reported brainstem-associated findings are specific to COVID-19 remains unknown.

Interestingly, intracapillary megakaryocytes have been reported in brain autopsies of COVID-19 patients (13), and were seen in 30% of COVID-19 cases in our study. Though previously hypothesized to be a potential contributor to COVID-19 related neurological complications, McMullen et al. reported that intracapillary megakaryocytes were not a specific marker of COVID-19, but instead seen in a multitude of acute lung pathologies (14). It seems unlikely that autopsy reports in the pre-pandemic era reported on this finding since they were only recently reported in literature (13). However, caution should be taken as this is a retrospective case-control study that may be biased to report more findings in COVID-19 samples.

COVID-19 ARDS patients had a higher frequency of vasculopathy noted on brain pathology in our study. COVID-19 has been associated with endothelial cell inflammation and vasculitis in both systemic and central nervous system (CNS) (15). On review of vasculopathy cases in our study, majority of vasculopathy was attributed to long term arteriosclerotic changes and no acute vascular changes were noted on pathology. While COVID-19 has been associated with CNS vasculitis in a few case reports, our study did not find any evidence of CNS vasculitis in COVID-19 ARDS patients.

One major limitation of our study was the difference in PMI time between COVID-19 and non-COVID-19 ARDS patients. Concern about the risk of exposure to COVID-19, particularly at the start of the pandemic, was likely the main factor for prolonged PMI in the COVID-19 group (16). Prolonged PMI may have resulted in degradation of brain tissue and underestimation of ABI (17). Our study only reviewed autopsy reports, slides were not revaluated, and further immunohistochemical staining for specific markers was not pursued for the purpose of the study. Though, it is standard for our pathologists to evaluate for inflammation on basic hematoxylin and eosin stains (H&E) and pursue further staining for specific markers if there is any concern. Intracapillary megakaryocytes in COVID-19 were also first identified on H&E and subsequent staining was only done to confirm their presence. While the process to evaluate all slides was similar, there may be a bias where pathologists were hypervigilant about any changes in COVID-19 patients. Findings of this study are limited to basic macro and microscopic examination of tissues given systematic restaining of slides was not pursued.

While this is one of the first matched case-control study evaluating ABI in COVID-19 ARDS patients, the study is limited by a small sample size and single center structure. We did see a trend of higher events of infratentorial ABI, ischemic infarcts, herniation, and edema in the COVID-19 group, though no established association with COVID-19 could be made. The small sample size can potentially miss certain brain pathologies related to COVID-19, so further assessment in a larger population is warranted.

## 5 Conclusions

Our study evaluating brain pathology between matched COVID-19 and non-COVID-19 ARDS patients did not show any differences in the frequency and patterns of ABI on basic histological examination. While the presence of intracapillary megakaryocytes was unique to COVID-19 in our study, further investigation in other cohorts for external validation is warranted.

## Data availability statement

The raw data supporting the conclusions of this article will be made available by the authors, without undue reservation.

## Ethics statement

Ethical review and approval was not required for the study on human participants in accordance with the local legislation and institutional requirements. Written informed consent from the patients/participants or patients/participants' legal guardian/next of kin was not required to participate in this study in accordance with the national legislation and the institutional requirements.

## Author contributions

MH: Conceptualization, Formal analysis, Methodology, Writing – original draft, Writing – review & editing. LZ: Data curation, Writing – review & editing. TZ: Data curation, Visualization, Writing – review & editing. NK: Data curation, Writing – review & editing. JS: Writing – review & editing. DH: Writing – review & editing. JT: Writing – review & editing. S-MC: Conceptualization, Supervision, Writing – review & editing, Resources.
